# Biocatalytic anti-Prelog reduction of prochiral ketones with whole cells of *Acetobacter pasteurianus* GIM1.158

**DOI:** 10.1186/1475-2859-13-84

**Published:** 2014-06-10

**Authors:** Peng-Xuan Du, Ping Wei, Wen-Yong Lou, Min-Hua Zong

**Affiliations:** 1 Laboratory of Applied Biocatalysis, South China University of Technology, Guangzhou 510640, China; 2 State Key Laboratory of Pulp and Paper Engineering, College of Light Industry and Food Sciences, South China University of Technology, Guangzhou 510640, China

**Keywords:** *Acetobacter pasteurianus* GIM1.158, Anti-Prelog, Asymmetric reduction, 2-octanone, (*R*)-2-octanol

## Abstract

**Background:**

Enantiomerically pure alcohols are important building blocks for production of chiral pharmaceuticals, flavors, agrochemicals and functional materials and appropriate whole-cell biocatalysts offer a highly enantioselective, minimally polluting route to these valuable compounds. At present, most of these biocatalysts follow Prelog’s rule, and thus the (*S*)-alcohols are usually obtained when the smaller substituent of the ketone has the lower CIP priority. Only a few anti-Prelog (*R*)-specific whole cell biocatalysts have been reported. In this paper, the biocatalytic anti-Prelog reduction of 2-octanone to (*R*)-2-octanol was successfully conducted with high enantioselectivity using whole cells of *Acetobacter pasteurianus* GIM1.158.

**Results:**

Compared with other microorganisms investigated, *Acetobacter pasteurianus* GIM1.158 was shown to be more effective for the reduction reaction, affording much higher yield, product enantiomeric excess (*e.e.*) and initial reaction rate. The optimal temperature, buffer pH, co-substrate and its concentration, substrate concentration, cell concentration and shaking rate were 35°C, 5.0, 500 mmol/L isopropanol, 40 mmol/L, 25 mg/mL and 120 r/min, respectively. Under the optimized conditions, the maximum yield and the product *e.e.* were 89.5% and >99.9%, respectively, in 70 minutes. Compared with the best available data in aqueous system (yield of 55%), the yield of (*R*)-2-octanol was greatly increased. Additionally, the efficient whole-cell biocatalytic process was feasible on a 200-mL preparative scale and the chemical yield increased to 95.0% with the product *e.e.* being >99.9%. Moreover, *Acetobacter pasteurianus* GIM1.158 cells were proved to be capable of catalyzing the anti-Prelog bioreduction of other prochiral carbonyl compounds with high efficiency.

**Conclusions:**

Via an effective increase in the maximum yield and the product *e.e.* with *Acetobacter pasteurianus* GIM1.158 cells, these results open the way to use of whole cells of this microorganism for challenging enantioselective reduction reactions on laboratory and commercial scales.

## Background

Enantiomerically pure chemicals are important building blocks for production of chiral pharmaceuticals, flavors, agrochemicals and functional materials [1]. For instance, (*R*)-2-octanol is a versatile intermediate for the synthesis of FLCD (ferroelectric liquid crystals FLCs) and several optically active pharmaceuticals such as steroid and insecticidal ectohormone. Asymmetric reduction of prochiral carbobyl compounds is an efficient method to produce chiral alcohols [2-5]. Whole-cell based biocatalytic reduction has attracted great attention and has been extensively investigated in recent years for the unique advantages such as outstanding enantioselectivity, mild reaction conditions, environmental friendliness and regeneration of cofactors *in situ*[6-8]. So far, yeasts, bacteria, fungi, and even plant tissues have been extensively researched as biocatalysts for bio-reduction processes [9,10], and many excellent biocatalytic reaction processes have been developed. However, most of these biocatalysts follow Prelog’s rule [11], and thus the (*S*)-alcohols are usually obtained when the smaller substituent of the ketone has the lower CIP priority. Only a few anti-Prelog (*R*)-specific whole cell biocatalysts have been reported [12-17]. As far as we know, most of the previously reported anti-Prelog microorganisms have not been used for industrial preparation of chiral alcohols for their relatively low catalytic activity and stereoselectivity. Take the substrate 2-octanone as example, the best reported result was given by a recombinant *Escherichia coli* overexpressing the genes of *a Lactobacillus brevis* alcohol dehydrogenase (LB-ADH) and a *Candida boidinii* formate dehydrogenase (CB-FDH) for cofactor regeneration [17]. In buffer system, the recombinant *E. coli* gave a very low yield of just 2% on a 1.4 mL scale, which increased to 55% when the bioreduction was on a 200 mL scale. Using ionic liquids (ILs) or organic solvents as a second phase could significantly increase the product yield [14,17-20]. For industrial application, the discovery of more efficient microorganisms would be of great significance.

On the other hand, *Acetobacter* is a kind of bacteria that is widely used for production of acetic acid owing to its large bioavailibility, easiness of treatment, less environmental pollution, low cost, and mild cultivation conditions [21]. Recently, *Acetobacter* sp*.* has been found to produce carbonyl reductase and shown catalytic activity for reduction of carbonyl compounds [22]. However, to our best knowledge, the use of *Acetobacter* sp*.* as a biocatalyst for the asymmetric reduction of prochiral ketones remains unexplored, with only few accounts published by us [16,20,23,24]. In these cases, a new acetic acid bacterium, *Acetobacter* sp. CCTCC M209061, was isolated from China kefir grains, and was capable of effectively catalyzing anti-Prelog asymmetric reduction of a number of carbonyl compounds with excellent enantioselectivity. Therefore, it can be well recognized that *Acetobacter* has the tremendous potential for asymmetric synthesis of valuable enantiopure alcohols.

In the present study, a number of microorganisms including acetic acid bacteria were tested for their potential for biocatalytic anti-Prelog asymmetric reduction of 2-octanone to (*R*)-2-octanol. Another *Acetobacter pasteurianus* GIM1.158 was found to be more active and enantioselective in catalyzing the bioreduction of 2-octanone and, for the first time, was applied as the biocatalyst for the asymmetric reduction of prochiral ketones. The effects of several crucial variables on the bioreduction of 2-octanone with whole cells of *Acetobacter pasteurianus* GIM1.158 were explored systematically. Also, the efficient biocatalytic process was evaluated on a preparative scale, and the applicability of the promising *Acetobacter pasteurianus* GIM1.158 was examined for the bioreduction of other prochiral ketones.

## Results and discussion

### Comparison of the biocatalytic enantioselective reduction of 2-octanone with *Acetobacter pasteurianus* GIM1.158 and other potential microorganisms

A variety of microorganisms have been reported to be efficient in catalyzing prochrial ketones to enantiomerically pure chrial alcohols [16,25-29]. Therefore, several commercial available microorganisms (*Acetobacter pasteurianus* GIM1.158, *Acetobacter* sp. CCTCC M209061, *Bacillus cereus* AS1.126, *Pseudomonas putica* GIM1.193*, Candida parapsilosis* CCTCCM203011*, Candia tropicalis* CICC1316, *Saccharomyces cerevisiae* GIM 2.34*, Rhodotorula* sp. AS2.2241, *Pseudomonas oleovorans* GIM1.304)were comparatively tested for their potential for the preparation of (*R*)-2-octanol via asymmetric reduction of 2-octanone in TEA-HCl buffer (50 mmol/L, pH 5.0). From the data summarized in Table 1, all of the nine tested strains, including five kinds of bacteria and four kinds of yeast cells, exhibited catalytic activity. Although these four yeasts were able to catalyze the asymmetric reduction of 2-octanone, they gave the undesired (*S*)-2-octanol. The varied stereoselectivity of these strains might be attributable to the expression of different reductases in the cells, which needs further investigation. Two *Acetobacter* strains, *Acetobacter pasteurianus* GIM1.158 and *Acetobacter* sp. CCTCC M209061 were capable of catalyzing the anti-Prelog stereoselective reduction of 2-octanone to (*R*)-2-octanol, and showed more activity and enantioselectivity compared to other microorganisms examined. *Acetobacter pasteurianus* GIM1.158 was slightly superior to *Acetobacter* sp. CCTCC M209061 in terms of the initial reaction rate, yield and product *e.e*. for the bioreduction of 2-octanone under the same reaction conditions. Besides, it was more easy and cheap to cultivate *Acetobacter pasteurianus* GIM1.158 cells. Obviously, *Acetobacter pasteurianus* GIM1.158 cell exhibited greater potential for efficient asymmetric reduction of 2-octanone to enantiopure (*R*)-2-octanol.

**Table 1:** Biocatalytic asymmetric reduction of 2-octanone with various strains

**Entry**	***Strain***	**V**_**0 **_**(×10**^**−1 **^ ***μ*** **mol/min)**	**Time (h)**	**Y **^***a ***^**(%)**	***e.e. *** **(%)**	***Config.***
1	*Acetobacter pasteurianus* GIM1.158	1.07	2.0	53.4	98.5	*R*
2	*Acetobacter* sp. CCTCC M209061	0.79	2.0	43.6	97.7	*R*
3	*Bacillus cereus* AS1.126	0.12	4.0	11.6	56.1	*S*
4	*Pseudomonas putica* GIM1.193	0.09	4.0	8.7	35.3	*S*
5	*Candida parapsilosis* CCTCCM203011	0.84	4.0	64.2	66.3	*S*
6	*Candia tropicalis* CICC1316	0.87	2.0	91.5	10.5	*S*
7	*Saccharomyces cerevisiae* GIM 2.34	0.45	2.0	45.4	>99.9	*S*
8	*Rhodotorula* sp. AS2.2241	0.55	2.0	54.7	22.3	*S*
9	*Pseudomonas oleovorans* GIM1.304	0.60	2.0	60.3	18.1	*S*

*Reaction conditions*: 2 mLTEA-HCl (50 mmol/L, pH 5.0), 25 mg/mL wet free cells, 10 mmol/L 2-octanone, 100 mmol/L isopropaanol, 30°C, 180 r/min.

Y^a^: The maximum yield.

### Effects of several key variables on the biocatalytic reduction of 2-octanone to (*R*)-2-octanol with *Acetobacter pasteurianus* GIM1.158 cells

To gain a deeper insight into the bioreduction and improve the results with respect to the initial reaction rate, the yield and the product *e.e.*, a systematic investigation was made of the effects of several important variables such as reaction temperature, buffer pH, different co-substrates and their concentration, substrate concentration, cell concentration and shaking rate on the reaction.

**Figure 1:**
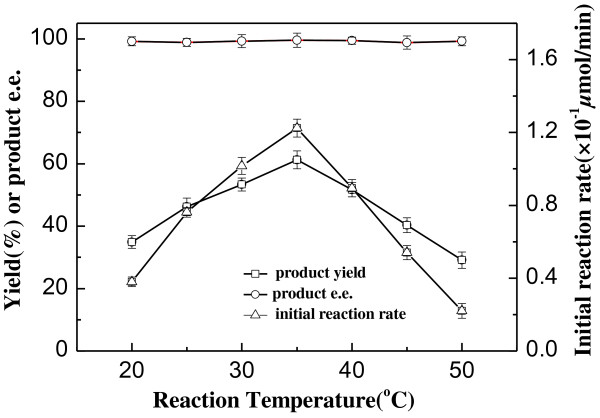
**Effect of reaction temperature on the asymmetric reduction of 2-octanone to *(R)*-2-octanol catalyzed by *Acetobacter pasteurianus *GIM1.158 cells**. Reaction conditions: 2 mL TEA-HCl buffer (50 mmol/L, pH 5.0), 10 mmol/L 2-octanone, 25 mg/mL wet cells, 100 mmol/L isopropanol, 180 r/min, various temperatures (20–50°C).

In general, temperature can affect the activity, the selectivity and the stability of a biocatalyst and the equilibrium of a reaction as well. Hence, the bioreduction was conducted at different reaction temperatures to see its impact on the reaction. As can be seen in Figure 1, within the examined temperature range between 20 and 50°C, both the initial reaction rate and the yield were affected markedly, while the product *e.e.* showed no significant variation and kept above 98.5%. The reaction accelerated substantially and the product yield increased with increasing temperature from 20 to 35°C. When the temperature was above 35°C, further increase in reaction temperature led to a clear drop in the initial reaction rate and the yield, which could be attributed to the partial inactivation of the biocatalysts at a higher temperature. Taking into account the initial reaction rate and yield, 35°C was considered as the optimum temperature for the bioreaction.

**Figure 2:**
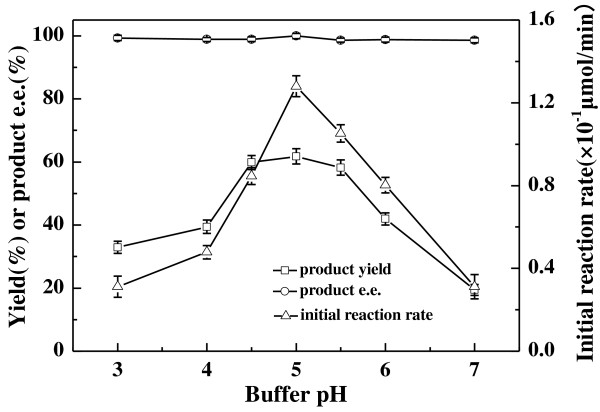
**Effect of buffer pH on the asymmetric reduction of 2-octanone to (*R*)-2-octanol catalyzed by *Acetobacter pasteurianus *GIM1.158 cells.** Reaction conditions: 2 mLTEA-HCl buffer (50 mmol/L, various pHs), 10 mmol/L 2-octanone, 25 mg/ml wet cells, 100 mmol/L isopropanol, 35°C, 180 r/min.

It is well known that pH play a crucial role in biocatalytic reactions. Variation of pH values could not only influence the activity and the selectivity of the biocatalyst, but also the regeneration of the coenzyme present in the microbial cells, which in turn affects the bioreduction rate markedly [30,31]. To our knowledge, buffer pH can alter the ionic state of the enzymes involved in the reaction and affect the local polarity of enzymes' active sites. As a result, an optimum pH value should exist for the bioreduction. As illustrated in Figure 2, buffer pH exerted a significant impact on both the initial reaction rate and the maximum yield of the reaction. Both of the reaction rate and the product yield increased with the rise of pH from 3.0 to 5.0. Further increasing buffer pH, however, resulted in a significant drop in product yield and initial reaction rate. Within the assayed buffer pH range from 3.0 to 7.0, there was only marginal change in the product *e.e*.. Obviously, the optimum buffer pH for the biocatalytic reaction was 5.0.

**Figure 3:**
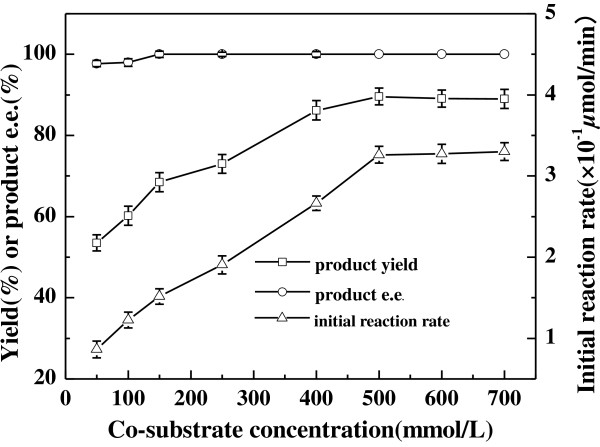
**Effect of co-substrate concentration on the asymmetric reduction of 2-octanone to (*R*)-2-octanol catalyzed by *Acetobacter pasteurianus *GIM1.158 cells.** Reaction conditions: 2 mL TEA-HCl buffer (50 mmol/L, pH 5.0), 10 mmol/L 2-octanone, 25 mg/ml wet cells, various concentrations of isopropanol, 35°C, 180 r/min.

Coenzyme recycling plays a special role in biocatalytic reduction reactions. The reductions could proceed smoothly with whole cells without adding expensive coenzyme (NADH or NADPH) only if a co-substrate is present for recycling the coenzyme [32]. Different co-substrates had been employed for the regeneration of coenzyme in some bioreduction reactions and varied largely in terms of the product yield and *e.e.*[27,33]. In this study, several commonly used co-substrates (glucose, glycerol, ethanol, isopropanol, methanol, fructose, sodium lactate) were tested to find the best one for the bioreduction of 2-octanone to (*R*)-2-octanol with *Acetobacter pasteurianus* GIM1.158 cells. As shown in Table 2, 2-octanone could be reduced to 2-octanol without adding any co-substrate (control), but the initial reaction rate and the maximum yield was very low (only 0.4 × 10^−2^ *μ*mol/min and 2%, respectively). Adding any single co-substrate markedly influenced the bioreaction. Among these 7 different co-substrates, ethanol gave the fastest reaction rate and the highest product yield while the product *e.e.* was relatively low (only 80.6%). It was obvious that glucose, sodium lactate and isopropanol could also be effectively utilized to regenerate coenzymes. The problem with the former two co-substrates was the low product *e.e.* (75.8% and 72.3%, respectively). Taken together, isopropanol gave the best result, with relatively high product yield (60.5%) and *e.e.* (above 99.9%). It is interesting to further investigate why the different co-substrates gave such an impact on the stereoselectivity of *Acetobacter pasteurianus* GIM1.158 cells. Additionally, increasing isopropanol concentration from 50 to 500 mmol/L gave rise to an increase in the initial reaction rate from 0.8 × 10^−1^ *μ*mol/min to 3.2 × 10^−1^ *μ*mol/min and the maximum yield from 52.5% to 89.1% (shown in Figure 3). The product *e.e.* kept constantly above 98.5% within the tested range of isopropanol concentration. It has been proved that *Acetobacter pasteurianus* GIM1.158 could tolerate high concentration of isopropanol up to 4 mol/L.

**Table 2:** **Effect of substrate concentration on the bioreduction of 2-octanone to (*R*)-2-octanol with *Acetobacter pasteurianus *** **GIM1.158 cells**

**Co-substrate**	**V**_**0 **_**(×10**^**−2**^ ***μ*** **mol/min)**	**Time (h)**	**Yield (%)**	***e.e. *** **(%)**
No cosubstrate	0.4	6.0	2.0	70.2
Glucose	4.4	6.0	41.9	75.8
Glycerol	1.8	6.0	18.7	8.6
Ethanol	15.5	2.0	82.4	80.6
Isopropanol	12.2	2.0	60.5	>99.9
Methanol	n.r.	6.0	n.r.	n.r.
Fructose	n.r.	6.0	n.r.	n.r.
Sodium lactate	13.8	2.0	72.4	72.3

*Reaction conditions*: 2 mL TEA-HCl buffer (50 mmol/L, pH 5.0), 25 mg/mL wet cells, 10 mmol/L 2-octanone, 100 mmol/L co-substrates, 35°C, 180 r/min.

**Figure 4:**
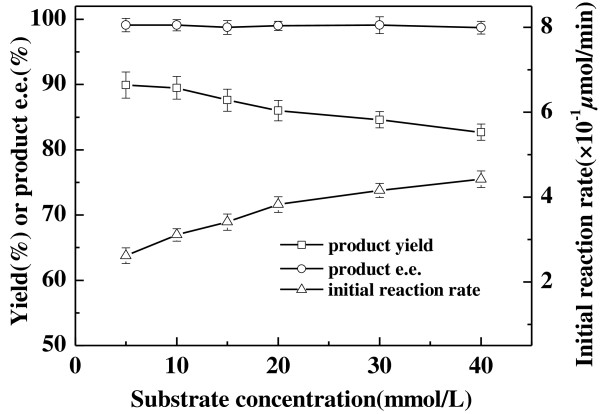
**Effect of substrate concentration on the asymmetric reduction of 2-octanone to (*R*)-2-octanol catalyzed by *Acetobacter pasteurianus *GIM1.158 cells.** Reaction conditions: 2 mL TEA-HCl buffer (50 mmol/L, pH 5.0), 500 mmol/L isopropanol, 25 mg/ml wet cells, various concentrations of 2-octanone, 35°C, 180 r/min.

As can be seen in Figure 4, the reaction accelerated and the maximum product yield showed no significant decrease when substrate concentration increased from 5 to 40 mmol/L, which was the maximum concentration of 2-octanone in buffer with the help of large amount solubilizer DMSO, suggesting no substrate inhibition in the tested range of substrate concentration. Throughout the range of substrate concentrations tested, the product *e.e.* showed no clear variation and remained above 98.5%. Obviously, the optimum concentration of 2-octanone was 40 mmol/L for the bioreduction with whole cell of *Acetobacter pasteurianus* GIM1.158 conducted in aqueous reaction system. It was noticed that *Acetobacter pasteurianus* GIM1.158 could tolerate fairly high concentrations of 2-octanone and 2-octanol. For the microbial reduction of 2-octanone, other microorganisms reported could generally tolerate relatively lower concentration of 2-octanone [34,35]. In the case of Baker's yeast used as biocatalyst for the bioreduction of 2-octanone [34], the yield of (*S*)-2-octanol decreased rapidly from 81% to 60% with increasing 2-octanone concentration from 2 to 10 mmol/L. For *Geotrchum candidum* IFO 4597, the most suitable concentration of 2-octanone was around 20 mmol/L and the obtained yield of (*S*)-2-octanol was about 62% [35]. Thus, *Acetobacter pasteurianus* GIM1.158 exhibited more promising and competitive for the biocatalytic asymmetric reduction of 2-octanone to (*R*)-2-octanol.

**Figure 5:**
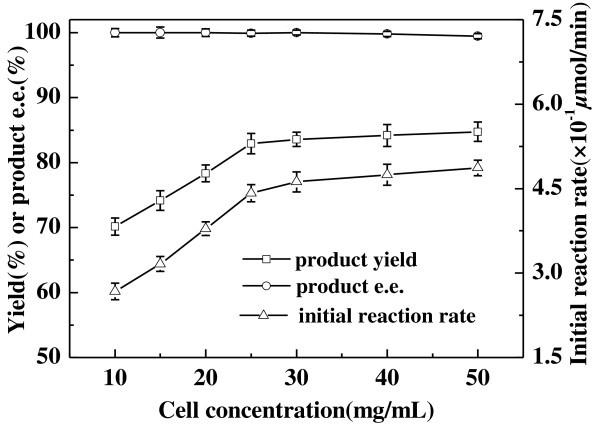
**Effect of biocatalyst concentration on the asymmetric reduction of 2-octanone to (*R*)-2-octanol catalyzed by *Acetobacter pasteurianus *GIM1.158 cells.** Reaction conditions: 2 mL TEA-HCl buffer (50 mmol/L, pH 5.0), 40 mmol/L 2-octanone, various concentrations of wet cells, 500 mmol/L isopropanol, 35°C, 180 r/min.

Cell concentration has not always been taken into account as a key factor for the whole cell-based biocatalytic processes. But we think it is an important factor to be considered in order to save biocatalyst as much as possible, making the bioreduction process more economically competitive. As shown in Figure 5, the initial reaction rate increased from 2.6 to 4.4 × 10^−1^ *μ*mol/min with increasing cell concentration from 10 to 25 mg/mL, while the maximum product yield from 70.6% to 82.9%. The product *e.e.* remained constant (above 98.5%) in the range of cell concentration. From an economic point of view, 25 mg/L *Acetobacter pasteurianus* GIM1.158 cells was enough for the bioreduction of 2-octanone to (*R*)-2-octanol.

**Figure 6:**
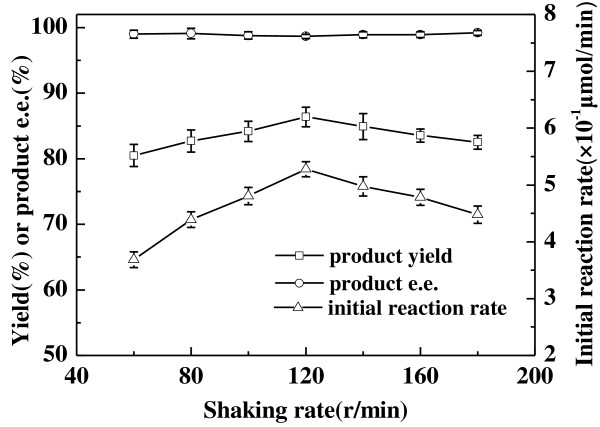
**Effect of shaking rate on the asymmetric reduction of 2-octanone to (*R*)-2-octanol catalyzed by *Acetobacter pasteurianus *GIM1.158 cells.** Reaction conditions: 2 mL TEA-HCl buffer (50 mmol/L, pH 5.0), 40 mmol/L 2-octanone, 25 mg/ml wet cells, 500 mmol/L isopropanol, 35°C, various shaking rates.

In general, shaking rate affects the diffusion of substrate and product in the reaction system, which would further affect the initial reaction rate and product yield. It was found that the reaction accelerated rapidly with increasing shaking rate from 60 to 120 r/min (Figure 6), with an obvious enhancement in the initial reaction rate from 3.6 to 5.2 × 10^−1^ *μ*mol/min, indicating that the mass transfer was the reaction rate limiting step. The optimum shaking rate was shown to be 120 r/min, above which little decrease in the initial reaction rate and the maximum yield was observed. Within the range of shaking rate tested, the product *e.e.* showed no significant alteration and remained above 98.5%.

Under the optimized reaction conditions described above (the optimal reaction temperature, buffer pH, co-substrate concentration, substrate concentration, cell concentration and shaking rate were 35°C, 5.0, 500 mmol/L isopropanol, 40 mmol/L, 25 mg/mL and 120 r/min, respectively), the biocatalytic asymmetric reduction of 2-octanone to (*R*)-2-octanol with *Acetobacter pasteurianus* GIM1.158 cells gave an encouraging result, with a yield of 89.5% and a product *e.e.* above 99.9% at a reaction time of 70 min.

### Biocatalytic anti-Prelog stereoselective reduction of various prochiral carbonyl compounds

In order to rationally evaluate the potential of *Acetobacter pasteurianus* GIM1.158 cells for the biocatalytic asymmetric reduction of carbonyl compounds, other nine prochrial carbonyl compounds (2-Pentanone, Pinacolone, 4'-Chloroacetophenone, 4'-Methyl-acetophenone, 4'-Hydroxyacetophenone, 3-Chloropropiophenone, Ethyl 4-chloroacetoacetate, Ethyl acetoacetate and Methyl acetoacetate) were also tested for the bioreduction conducted in TEA-HCl buffer. As listed in Table 3, *Acetobacter pasteurianus* GIM1.158 was able to catalyze the anti-Prelog asymmetric reductions of all the tested ketones to the corresponding alcohols with high enantioselectivity, and the achieved product *e.e.* reached more than 92.0%. When the substrates were aliphatic ketones, the yield and the product *e.e.* increased with the side-chain of the carbonyl group. In the case of prochiral keto esters, the microbial cells exhibited high catalytic activity and enantioselectivity. For example, the obtained yield of ethyl (*R*)-3-hydroxybutyrate with *Acetobacter pasteurianus* GIM1.158 was slightly higher than that with *Acetobacter* sp. CCTCC M209061 in aqueous reaction system (85.8% *vs* 82.6%) [36]. Besides, *Acetobacter pasteurianus* GIM1.158 was more active and effective in enantioselectively catalyzing the bioreduction of methyl acetoacetate to methyl (*R*)-3-hydroxybutyrate, which was not reported previously. When the tested substrates were aromatic ketones, apart from 4'-chloroacetophenone, the achieved yield (more than 94.9%) and the product *e.e.* (more than 97.1%) were very satisfactory. In particular, the biocatalytic asymmetric reduction of 3-chloropropiophenon to (*S*)-3-chloro-1-phenylpropanol was successfully conducted using *Acetobacter pasteurianus* GIM1.158 cells with high product yield (94.9%) and product *e.e.* (99.7%), which were superior to those with *Candida utilis* reported previously (yield: 85%; product *e.e.*: 99.5%) [37]. The above-described results clearly showed that the established biocatalytic system with *Acetobacter pasteurianus* GIM1.158 cells can also be successfully used for efficient synthesis of other important chiral alcohols.

**Table 3:** **Biocatalytic anti-Prelog stereoselective reduction of various prochiral carbobyl compounds with *Acetobacter pasteurianus *** **GIM1.158 cells**

**Prochiral ketones**	**Structure**	**V**_**0 **_**(×10**^**−1**^ ***μ*** **mol/min)**	**Yield (%)**	***e.e.. *** **(%)**	***Config.***
2-Pentanone		9.78	81.5	92.0	*R*
Pinacolone		11.3	94.5	95.2	*R*
4'-Chloroacetophenone		7.3	60.4	>99.9	*R*
4'-Methyl-acetophenone		11.9	99.5	99.6	*R*
4'-Hydroxyacetophenone		11.7	97.8	97.1	*R*
3-Chloropropiophenone		5.7	94.9	99. 7	*S*
Ethyl 4-chloroacetoacetate		14.9	74.4	97.3	*S*
Ethyl acetoacetate		17.2	85.8	98.7	*R*
Methyl acetoacetate		18.6	92.9	97.2	*R*

*Reaction conditions*: 2 mL TEA-HCl buffer (50 mmol/L, pH 5.0), 25 mg/mL wet cells, 5 mmol/L various prochiral ketones, 500 mmol/L isopropanol, 35°C, 120 r/min, reaction time 2 h.

### Preparative scale bioreduction of 2-octanone to (*R*)-2-octanol

Biotransformation on a 200-mL scale was performed to determine scalability of biocatalytic asymmetric reduction of 2-octanone to (*R*)-2-octanol with *Acetobacter pasteurianus* GIM1.158 cells. The reaction process was monitored by GC analysis and the product was extracted from the reaction mixture with acetic ether when no more substrate was converted to the product. A final chemical yield of 95.0% was achieved and the product *e.e.* was above 99.9% in 70 min. To our best knowledge, the reported maximum yield of biotransformation of 2-octanone to (*R*)-2-octanol in buffer was only 55% while the highest substrate concentration was much lower [17].

It should be emphasized that the bioreduction process described above suffered from the drawback of low substrate concentration and overall productivity for the large-scale industrial application because of the low solubility of 2-cotanone in buffer system. We believe that the reaction efficiency could be further improved by employing a biphasic system containing an organic solvent or preferably a biocompatible ionic liquid to relieve the restriction of low solubility of substrate in buffer as the concentration of 2-octanone in the second phase could reach up to 1.5 mol/L.

## Conclusions

The preparation of enantiopure (*R*)-2-octanol on a 200-mL preparative scale can be successfully conducted through anti-Prelog asymmetric bioreduction of 2-octanone with *Acetobacter pasteurianus* GIM1.158 cells. Under the optimal conditions (35°C, pH 5.5, 500 mmol/L isopropanol as co-substrate, substrate concentration 40 mmol/L, cell concentration 25 mg/L, shaking rate 120 r/min), the maximum yield and the product *e.e.* were 95.0% and above 99.9% respectively in 70 min. Furthermore, *Acetobacter pasteurianus* cells exhibited high catalytic activity for highly enantioselective reduction of various kinds of carbonyl compounds.

## Material and methods

### Biological and chemical materials

*Acetobacter pasteurianus* GIM1.158 was purchased from Guangdong Culture Collection Center. Other strains (*Acetobacter* sp. CCTCC M209061, *Bacillus cereus* AS1.126, *Pseudomonas putica* GIM1.193*, Candida parapsilosis* CCTCCM203011*, Candia tropicalis* CICC1316, *Saccharomyces cerevisiae* GIM 2.34*, Rhodotorula* sp. AS2.2241, *Pseudomonas oleovorans* GIM1.304) used in this work were kept in our laboratory (Lab of Applied Biocatalysis, South China University of Technology, China).

2-Octanone (99% purity) and ethyl acetoacetate were purchased from Alfa Aesar (USA). (*R*)-2-Octanol (98% purity) and (*S*)-2-octanol (98% purity) were from Sigma-Aldrich (USA). Other prochiral ketones and the corresponding alcohols were obtained from Aldrich-Fluka and were all over 97% purity. All other chemicals were from commercial sources and were of analytical grade.

### Cell cultivation

*Acetobacter pasteurianus* GIM1.158 cells were cultivated on medium containing 6 g/L yeast extract, 6 g/L peptone, 10 g/L sodium lactate solution, 0.75 g/L K_2_HPO_4_, 0.5 g/L NaH_2_PO_4_, 0.1 g/L MnSO_4_, 0.2 g/L MgSO_4_, 0.1 g/L CaCl_2_. Other bacteria cells were grown in Nutrient Broth Medium (NB). Yeast cells were cultivated in Yeast Extract Peptone Dextrose Medium (YPD).

### General procedure for biocatalytic asymmetric reductions of prochiral ketones

In a typical experiment, 2.0 mL of TEA-HCL buffer (pHs 3.0-7.0, 50 mmol/L) containing wet cells (10–50 mg/mL) and a predetermined quantity of co-substrate (50–700 mmol/L) were added to a10-mL Erlenmeyer flask capped with a septum, and pre-incubated in a water-bath shaker at a specified shaking rate (60–180 r/min) and an appropriate temperature (20–50°C) for 15 min. The reaction was initiated by adding various prochial ketones (5–40 mmol/L) to the mixture. Aliquots (20 μL) were withdrawn at specified time intervals. The product and the residual substrate were extracted with ethyl acetate (40 μL) for twice containing 5.0 mmol/L *n*-decane (as internal standard) prior to GC analysis. Details about reaction temperature, buffer pH, substrate concentration, co-substrate concentration, cell concentration and shaking rate are specified for each case.

### Preparative scale biocatalytic reduction of 2-octanone to (*R*)-2-octanol

The preparative scale biocatalytic reduction of 2-octanone with whole cells of *Acetobacter pasteurianus* GIM1.158 was performed under the optimized reaction conditions. The bioreduction reaction was conducted by adding 5 g wet cells of *Acetobacter pasteurianus* (25 mg/mL) and 8 mmol of 2-octanone (40 mmol/L) to 200 mL of TEA-HCl buffer (50 mmol/L, pH 5.0) containing 500 mmol/L isopropanol as co-substrate at 35°C and 120 r/min. The reaction was terminated when no substrate was transformed to product any more. Then the product and the residual substrate were extracted with ethyl acetate (2 × 200 mL). The yield and *e.e.* of (*R*)-2-octanol were determined by GC analysis.

### GC analysis

The reaction mixture was analyzed by a Shimadzu GC-2010 with a flame ionization detector and a HP-chiral CB column (30 m × 25 mm × 0.25 m) (USA). The split ratio was 50:1. The injector and the detector were both at 250°C. The carrier gas was nitrogen (>99.9). 2-Octanol was derived with trifluoroacetic anhydride before GC analysis. The column temperature was held at 110°C and the flow rate of nitrogen was 0.75 mL/min. The retention times for derived 2-octanol, *n*-decane and 2-octanone were 3.9, 4.1 and 4.7 min. For the determination of the product *e.e.*, the column temperature was kept at 85°C for 15 min while the flow rate of nitrogen in the column was 0.5 mL/min. The retention times for (*S*)-2-octanol and (*R*)-2-octanol were 12.28 and 12.51 min, respectively. For other substrates and the corresponding products, the column temperature, the flow rate of nitrogen in the column and the retention times were as follows.

2-Pentanone/2-pentanol, 3,3-dimethyl-2-butanone/3,3-dimethyl-2-butanol: 80°C, 13.5 min, 60°C/min to 145°C, retention times: derivatized (*S*)-2-pentanol (11.1 min), derivatized (*R*)-2-pentanol (11.4 min), 2-pentanone (16.2 min); derivatized (*S*)-2-(3,3-dimethyl)butanol (9.6 min), derivatized (*R*)-2-(3,3-dimethyl)butanol (9.9 min), 3,3-dimethyl-2-butanone(15.7 min). 4'-Methoxyacetophenone/1-(4'-methoxyphenyl) ethanol, 4'-chloroacetophenone/1-(4'-chlorophenyl) ethanol, 4'-hydroxyacetophenone/1-(4'-hydroxyphenyl)ethanol: 140°C (10 min), 1°C /min to 145°C (4 min), retention times: 4-methoxyacetophen-one (11.6 min), (*R*)-1-(4'-methoxyphenyl)ethanol (15.2 min), (*S*)-1-(4'-methoxyphenyl)ethanol (15.5 min); 4'-chloroacetophenone (8.3 min), (*R*)-1-(4'-chlorophenyl)ethanol (12.2 min), (*S*)-1-(4'-chlorophenyl)ethanol (12.6 min); 4'-hydroxyacetophenone(10.1 min), (*S*)-1-(4'-hydroxyphenyl)ethanol (14.7 min), (*R*)-1-(4'-hydroxyphenyl)ethanol (15.1 min).

3-Chloropropiophenone/3-chloro-1-phenylpropanol: 140°C, 30 min, retention times: 3-chloropropiophenone (16.0 min), (*R*)-3-chloro-1-phenylpropanol (26.5 min), (*S*)-3-chloro-1-phenylpropanol (26.9 min). Ethyl 4-chloroacetoacetate/ethyl −4-chloro-3-hydroxybutyrate, ethyl acetylacetate/ethyl 3-hydroxybutyrate, methyl acetoacetate/methyl −3-hydroxybutyrate: 80°C (20 min), 50°C/min to 155°C (6 min), retention times: ethyl 4-chloroacetoacetate (24.2 min), derivatized ethyl (*R*)-4-chloro-3-hydroxybutyrate (19.1 min), derivatized ethyl (*S*)-4-chloro-3-hydroxybutyrate (19.4 min); ethyl acetylacetate (23.2 min), derivatized ethyl (*R*)-3-hydroxybutyrate (17.3 min), derivatized ethyl (*R*)-3-hydroxybutyrate (17.6 min); methyl acetoacetate (21.1 min), derivatized methyl (*R*)-3-hydroxybutyrate(15.2 min), derivatized methyl (*R*)-3-hydroxybutyrate (15.4 min).

## Competing interests

The authors declare that they have no competing interests.

## Authors’ contributions

P-XD and PW conducted the experiments. P-XD wrote the main text, figures and tables. W-YL and M-HZ corrected and helped to draft the manuscript. All authors have read and approved the final manuscript.
